# Adapting and scaling a proven diabetes prevention program across 11 worksites in India: the INDIA-WORKS trial

**DOI:** 10.1186/s43058-023-00516-1

**Published:** 2023-11-13

**Authors:** Mary Beth Weber, Elizabeth C. Rhodes, Harish Ranjani, Panniyammakal Jeemon, Mohammed K. Ali, Monique M. Hennink, Ranjit M. Anjana, Viswanathan Mohan, K. M. Venkat Narayan, Dorairaj Prabhakaran

**Affiliations:** 1 Emory Global Diabetes Research Center, Woodruff Health Sciences Center, Emory University, Atlanta, GA USA; 2 Hubert Department of Global Health, Rollins School of Public Health, Emory University, Atlanta, GA USA; 3 Madras Diabetes Research Foundation/Dr. Mohan’s Diabetes Specialities Centre, Chennai, India; 4 Achutha Menon Centre for Health Science Studies, Sree Chitra Tirunal Institute for Medical Sciences and Technology, Trivandrum, India; 5 Department of Family and Preventive Medicine, School of Medicine, Emory University, Atlanta, GA USA; 6 Public Health Foundation of India, New Delhi, India; 7 Centre for Chronic Disease Control, New Delhi, India

**Keywords:** Implementation, Adaptation, Worksites, India, Cardiometabolic disease, Diabetes, Prevention, Qualitative research, Structured lifestyle modification

## Abstract

**Background:**

Structured lifestyle change education reduces the burden of cardiometabolic diseases such as diabetes. Delivery of these programs at worksites could overcome barriers to program adoption and improve program sustainability and reach; however, tailoring to the worksite setting is essential.

**Methods:**

The Integrating Diabetes Prevention in Workplaces (INDIA-WORKS) study tested the implementation and effectiveness of a multi-level program for reducing cardiometabolic disease risk factors at 11 large and diverse worksites across India. Herein, we describe and classify program adaptations reported during in-depth interviews and focus group discussions with worksite managers, program staff, and peer educators involved in program delivery, and program participants and drop-outs. We used thematic analysis to identify key themes in the data and classified reported program adaptations using the FRAME classification system.

**Results:**

Adaptations were led by worksite managers, peer educators, and program staff members. They occurred both pre- and during program implementation and were both planned (proactive) and unplanned (proactive and reactive). The most frequently reported adaptations to the individual-level intervention were curriculum changes to tailor lessons to the local context, make the program more appealing to the workers at the site, or add a wider variety of exercise options. Other content adaptations included improvements to the screening protocol, intervention scheduling, and outreach plans to tailor participant recruitment and retention to the sites. Environment-level content adaptations included expanding or leveraging healthy food and exercise options at the worksites. Challenges to adaptation included scheduling and worksite-level challenges. Participants discussed the need to continue adapting the program in the future to continue making it relevant for worksite settings and engaging for employees.

**Conclusion:**

This study describes and classifies site-specific modifications to a structured lifestyle change education program with worksite-wide health improvements in India. This adds to the literature on implementation adaptation in general and worksite wellness in India, a country with a large and growing workforce with, or at risk of, serious cardiometabolic diseases. This information is key for program scale-up, dissemination, and implementation in other settings.

**Trial registration:**

Clinicaltrials.gov NCT02813668. Registered June 27, 2016

Contributions to the literature

- This paper describes adaptations to an evidence-based lifestyle change education program for prevention of diabetes and other cardiometabolic diseases at eleven large and diverse worksites in India. India has the second largest workforce in the world and has a large population at risk of developing conditions like type 2 diabetes and high blood pressure at younger ages.
- To successfully implement programs shown to be effective in research studies for population-wide impact, scientists, clinicians, and community leaders need to understand how these programs were adapted to the needs of the target population. This study provides guidance for culturally adapting a structured lifestyle change education program at worksites in India and could assist in program implementation in other similar settings.
- Implementation research is a growing field of study; however, there are limited studies that systematically report program modifications and adaptations, a key factor in successful implementation. This paper reports on the adaptations made to the INDIA-WORKS implementation trial at 11 worksites across India and categorizes and describes them using the Framework for Reporting Adaptations and Modifications-Enhanced (FRAME).
- Our study adds to the limited literature using FRAME in a low- and middle-income country context. In the discussion, the study team reflects on the utility and challenges of using this tool in the context of Indian worksites.

## Background/introduction

At least 101 million and 136 million people in India have diabetes or prediabetes, respectively, many of whom are working age adults [1]. Diabetes profoundly impacts health systems (e.g., resources for care), individual and family finances (e.g., care costs, loss of wages due to lost work time), individuals (e.g., debilitating secondary complications, lower quality of life), and worksites (e.g., lower productivity, lost work time) [2–5]. Lifestyle interventions are structured education programs for weight loss, increasing healthy lifestyle behaviors, improving cardiovascular risk factors, delaying the onset of diabetes in at-risk individuals, and/or preventing secondary complications and reducing medication use in individuals with diabetes [6–16]. Lifestyle interventions have been shown to be efficacious and effective [17–20]. However, real-world implementation is challenging because existing programs require large upfront investments of money and staff and participant time with returns on investments only recouped over years [21].

Delivering lifestyle interventions at worksites, using the existing structure of worksite health facilities for testing and utilizing employees as peer educators, could be an effective and cost-effective approach for delivering lifestyle change education. Furthermore, it may overcome many individual-level barriers to participation in a lifestyle change program (e.g., lack of time and social support, inability to locate resources) and could be beneficial to employers (e.g., higher employee satisfaction and retention; less lost productivity) [22–27]. To be successfully implemented and scaled, evidence-based interventions need to be adapted to succeed in the context where delivered [28]. Translation of evidence-based interventions to the broader population requires adaptations for delivery in community settings that lower financial and personnel costs and increase the acceptability and appropriateness of the program for participants, cost-effectiveness for payers, and program scalability and sustainability. Program modifications are changes made to interventions either deliberately or in response to challenges arising during implementation. Adaptations, a type of program modification, are deliberate program changes made to improve the program’s update, delivery, effectiveness, or acceptability in a particular context [29, 30]. Documenting these adaptations, particularly those that occur after program implementation begins, is crucial to understanding how to adapt real-world programs across settings [31, 32]. Exploring these program changes will provide key information for others seeking to implement worksite-based interventions for cardiometabolic conditions, particularly in India, where there is a large and growing need for effective, community-based programming to overcome barriers to chronic disease prevention.

Herein we report on the adaptations made during the Integrating Diabetes Prevention in Workplaces (INDIA-WORKS, Clinicaltrial.gov, NCT02813668, registered on June 27, 2016, https://classic.clinicaltrials.gov/ct2/show/NCT02813668) implementation study. INDIA-WORKS aimed to overcome barriers to worksite delivery of lifestyle interventions by using the peer educator model while promoting worksite-based environmental changes to support program participants. We applied the FRAME [30] classification system, a tool for documenting and categorizing program modifications and adaptations in implementation programs and research studies, to describe program changes across worksites.

## Methods

### Study setting—the INDIA-WORKS study

The INDIA-WORKS intervention was based on the lifestyle education program tested in the Diabetes Community Lifestyle Improvement Program (D-CLIP) trial [33]. D-CLIP was a randomized controlled translation research study testing the effectiveness, acceptability, and cost-effectiveness of a lifestyle change curriculum based on the U.S. Diabetes Prevention Program (DPP) [14, 34] plus the addition of metformin as needed for reducing diabetes risk in overweight or obese Indian adults with pre-diabetes. The D-CLIP curriculum included 4 months of weekly, group-based classes on making healthy dietary changes, increasing physical activity, building social support, and overcoming barriers followed by 2 months of classes on maintaining lifestyle changes. The D-CLIP intervention reduced diabetes incidence and improved cardiometabolic risk factors [17]. The D-CLIP curriculum, including lesson plans, handouts, and coach scripts, were modified for the INDIA-WORKS program as described below and in the results. INDIA-WORKS participants were not provided metformin as part of the study. The INDIA-WORKS study sought to test the application of the D-CLIP program for broader prevention of cardiovascular disease by delivering the program to individuals with both prediabetes and unmedicated diabetes.

INDIA-WORKS was a pre-post design, implementation research study conducted at 11, medium to large worksites (1500–50,000 employees) across ten companies in South, Central, and East India. Worksites were diverse and included mineral-based and product manufacturing, energy, technology, and transportation industry worksites. All sites included a variety of workers in manual labor and office-based positions. Inclusion criteria for employees to participate in the intervention classes were age ≥ 18 years; overweight or obese using World Health Organization defined South-Asian cut-points: BMI ≥ 23 to < 27.5 kg/m^2^ (overweight), BMI ≥ 27.5 kg/m^2^ (obese), and/or waist circumference ≥ 90 cm for men and ≥ 80 cm for women [35]; have prediabetes (HbA_1c_ of 5.7–6.4%) or diabetes (HbA_1c_ ≥ 6.5%); not currently taking any diabetes medications; not pregnant or breastfeeding; and without history of heart disease, current serious illness, or conditions impeding participation in an unsupervised physical activity and diet change program. Eligible individuals were identified using a two-phased screening program at each worksite. Multi-stage screening activities were conducted over multiple days at the worksite to minimize time away from work for employees and avoid unnecessary testing for ineligible individuals. Study testing was conducted in collaboration with worksite health clinics, which are staffed by nurses and physicians who provide routine healthcare for the workers at each site. Individuals with newly identified or uncontrolled disease (e.g., diabetes, hypertension or dyslipidemia) were referred to the onsite physicians.

The multi-level intervention package included both a structured lifestyle change program (individual-level) and supportive changes to the worksite environment (environmental-level). The individual-level intervention was designed to benefit employees enrolled in the lifestyle change program, while work environment changes could benefit all employees. The intervention package included core components which remained standard across sites and adaptable elements which could be modified to suit individual worksite settings. Core components were (1) key design features of the DPP lifestyle change program [34], the basis for D-CLIP and subsequently the INDIA-WORKS curriculums; (2) features shown to be important for user acceptability in the D-CLIP study; or (3) components recommended by worksite advisors during program planning. Table 1 describes the planned core components and adaptable elements of the intervention package.

**Table 1:** INDIA-WORKS planned core components and adaptive elements

**Aspect of the intervention**	**Core** c**omponents**	**Adaptive** e**lements**
**Lifestyle education program**		
Class format	16 weekly hour-long core sessions plus 8 monthly maintenance sessions based on the curriculum developed for the D-CLIP and DPP studies. Order of lessons is set and should not be adapted. Individual lessons include a mix of teaching/learning styles including didactic instruction, role playing, group discussions, and question and answer sessions.	Timing of classes Composition of class cohorts
Lifestyle change curriculum	Educational curriculum focused on diet improvement (increasing fiber and fruit and vegetable intake, decreasing intake of high fat and high carbohydrate foods, portion sizes), increasing physical activity, decreasing stress and dealing with stress in a healthy way, and maintaining healthy behaviors	Most classes included substantial time for group activities and discussions which could be tailored to the needs of the sites and individual classes
Physical activity education	All participants provided with training on starting and increasing exercise, exercise safety, overcoming barriers, and increasing activities of daily living. Participants were taught a simple routine of stretches and strength training using their own body weight.	Sites with exercise facilities and in-house gym staff could provide more extensive exercise training. Worksites in or near Chennai, India provided a detailed 16 week physical activity curriculum to their participants and it was delivered by certified trainers.
Class instructors	Education team made up of trained peer educators and professional health educators	Although all peer educators were encouraged and empowered to lead lifestyle classes, the balance of teaching between the peer educator and the professional health educator could vary by site.
Additional support to intervention class participants	SMS text messages delivered weekly reinforcing lessons taught during lifestyle classes after the core classes Food and activity diaries to support and track changes. Weight, waist circumference, and blood pressure tracking. Pedometer step counts. Tools for sharing information and peer support.	Information or support could be shared via different channels (e.g., listserv, WhatsApp Messenger, in person)
Participant goals	All participants given two goals to achieve during the intervention: (1) increase physical activity to at least 150 min per week of moderate level activity and (2) lose at least 5% of their baseline body weight (via diet and activity changes).	Participants were given choices for how to reach study goals and then could work towards these goals choosing the tools that are right for them individually.
**Worksite health promotion efforts**		
Canteen changes	Worksites encouraged and supported in making positive changes to the types of foods and serving sizes in the company canteens	There was flexibility in what healthy foods the canteen will offer, and the type and extent of changes made depend on capacity and resources at the worksite
Health screening	Health screenings offered to all interested employees.	Health screens could be offered in a way that best suited the worksite (e.g., single day screening events for all workers, scheduled visits for individual workers, etc.)
Physical activity promotion	Walking groups at the worksites open to all employees.	Activation of dormant gyms or activity areas at worksites Sites supported in making other changes to promote more active choices among employees including informational signage, designating areas for walking or other activities, and proving exercise training on site.

### Data collection

Adaptations to the INDIA-WORKS curriculum and delivery were identified and described through qualitative interviews (individual in-depth interviews [IDIs] and focus group discussions [FGDs]) with individuals involved in the delivery, management, and use of the INDIA-WORKS program. IDIs explored the individual level experiences of high-level worksite managers (*n* = 16), peer educators (*n* = 29), program drop-outs (*n* = 29), and INDIA-WORKS team members involved in on-site program implementation (*n* = 8). FGDs (*n* = 14) with program participants who completed the program described community-level experiences of the program. IDI and FGD guides covered program views, context-specific program adaptations and their challenges, and suggestions for sustaining the program. Qualitative data collection occurred during the first year of the INDIA-WORKS study (December 2018–June 2019). IDI/FGD participants were recruited via a gatekeeper strategy. We first discussed the purpose and data collection procedures with managers and requested their permission and support for the data collection. We included participants suggested by managers as well as individuals identified by local study staff in our pool of potential interviewees to ensure a diverse and information-rich sample for the qualitative data. Potential IDI/FGD participants were approached via email, phone call, and/or in-person contact.

IDIs/FGDs were conducted privately by a trained member of the INDIA-WORKS study team at a private location at the participant’s worksite in their preferred language (English, Hindi, Malayalam, or Tamil). Participants participated in only one FGD or IDI. For manager interviews and INDIA-WORKS staff interviews, interviewers had been in contact with the interviewees; however, care was taken to ensure that interviews with staff were not done by that person’s supervisors. For all other IDIs/FGDs, there was no prior relationship between the interviewer and interviewees. Participants provided written informed consent for the interview and audio-recording of the discussion; as part of the consent process, the goals of the qualitative data collection and reasons for the research were described to participants. Interview team training covered interview and FGD moderation techniques, reflexivity, and the data collection tools. The interview team met at least once per week to review data quality and assess data saturation. Saturation was determined when (1) there was sufficient diversity in participants recruited and experiences shared; (2) different types of worksites were included; and (3) data saturation was observed, whereby the interview team assessed that no new issues were being reported. When these requirements were fulfilled, code saturation was reached and data collection ceased [36].

The discussions were guided using semi-structured IDI/FGD guides, which were informed by the D-CLIP study [33] and Proctor’s framework [37, 38] and made culturally appropriate for the target individuals and setting. Guides were pilot tested internally by conducting practice interviews with team members and in the field in each language, refined, and translated before study use. FGDs and IDIs audios were digitally-recorded, transcribed verbatim, and translated into English. Translated transcripts were checked for accuracy of translation. To enable comparisons between subsets of those interviewed (e.g., different sexes), demographic information was collected.

### Data analysis

Translated, verbatim transcripts of the IDIs and FGDs were managed using MAXQDA 2020 (VERBI Software). Using thematic analysis techniques, a codebook including both deductive and inductive codes for each set of qualitative data (e.g., peer educator IDIs, FGDs with program participants) was created. Three coders coded the data which involved assessing inter-coder agreement, rectifying discrepancies in coding, and then coding transcripts independently. Four codes, “Adaptations”, “IW Worksite Context”, “Sustainability”, and “Future Suggestions/Adaptations” described program adaptations and detailed suggested adaptations for the future. These codes were the focus of this analysis.

Coded segments were reviewed and a thick description of each code was developed describing adaptations to the INDIA-WORKS curriculum, worksite environmental changes, changes to improve program acceptability, challenges to program adaptation, and suggestions for additional changes for the future. Modifications and adaptations were then categorized and described by applying the FRAME [30], a comprehensive tool for describing programmatic adaptations in implementation science. The FRAME categorizes adaptations and modifications using the following domains:

1. What was modified
2. The level of the modification—here, individual-level or environmental-level
3. The nature, or type, of the modification—e.g., tailoring, refining, adding elements
4. The goal of and reason for the modification
5. When the modification was made—pre-implementation or during implementation
6. If the modification was planned or unplanned and proactive or reactive
7. Who participated in the decision to make the modification—here, reported as the specific players, INDIA-WORKS staff, worksite managers, and peer educators, which are included in the following FRAME categories for this domain: INDIA-WORKS staff includes treatment/intervention team, program developers and purveyors, and individual practitioners/facilitators; peer educators are individual practitioners/facilitators; and worksite managers are administration
8. If the modification was fidelity consistent

In addition, we described challenges to adapting the program at the worksite and suggestions for future program adaptations. Results were shared and discussed with members of the study team involved in field work; due to challenges of the COVID-19 pandemic at the worksites, it was not feasible to review findings and interpretations with worksite interviewees.

## Results

Program adaptations were made at worksites before and during the first year of project implementation. Worksite managers, peer educators, and INDIA-WORKS staff members all reported making program adaptations to ensure successful program delivery at individual worksites. Adaptation were fidelity-consistent, unless otherwise noted, reflecting modifications to adaptable, and not core, components of the intervention package. Herein, we report on cohort-level adaptations to the individual-level intervention content including adaptations to study curriculum or testing and those made at the environment-level and affecting all participants at that site. For each, we map the adaptations to FRAME components (Tables 2 and 3), providing illustrative examples in the text. In addition, we report on adaptations to training.

**Table 2:** Content-level adaptations to the INDIA-WORKS program

**Description**	**Examples**	**Nature**^**a**^**/additional detail**	**Goal** **Reason**^**b**^	**Timing**^**c**^		**Planned**^**d**^			**Players**^**e**^		
				**Pre-implementation**	**Implementation**	**Planned/proactive**	**Planned/reactive**	**Unplanned/reactive**	**INDIA-WORKS staff**	**Worksite managers**	**Peer educators**
Curriculum adaptations: format changes to class scripts and handouts	- Translation to local language	Tailoring/make materials more understandable and approachable for users	Address cultural factors Recipient level: first/spoken language	X		X			X	X	
	- Use less technical language - Less formal presentation style	Tailoring/make materials more understandable and approachable for users	Improve fit with recipients Recipient level: literacy and education level		X	X	X		X		X
	- Focus on local foods in lessons	Tailoring/make materials more approachable for users; in some cases based on views of peer educators	To address cultural factors Sociopolitical/outer context: societal/cultural norms		X	X		X			X
Curriculum adaptations: additions to curriculum	- Addition of more hands-on/interactive elements in class - Added training on physical activity skills	Adding elements/make materials more understandable and approachable for users	Improve fit with recipients Recipient level: motivation and readiness		X	X			X		X
	- Adding different and more advanced exercise classes	Adding elements/make program more appealing to users	Increase retention Recipient level: motivation and readiness		X	X		X		X	X
	- Encouraging participants to use existing exercise spaces (trails, gyms) - Encouraging participants to use factory/office spaces and grounds for walking - Encourage use of health food canteens	Tailoring: guiding participants to leverage worksite resources	Improve effectiveness/outcomes Organizational level: available resources Recipient level: motivation and readiness		X	X	X		X	X	X
Study testing protocol adaptations	- Doing all study testing at a single visit minimized drop-out - Adding additional testing of interest to the worksite	Tailoring: conducting study testing to reduce drop-outs and acceptability	Improve retention Recipient level: motivation and readiness		X			X	X	X	
Class logistics	- Holding classes at times convenient to workers at site - Selecting class venues to maximize participation - Allowing classes to be held during working hours	Tailoring/planning classes to balance needs of workers and the worksite	Improve feasibility Recipient level: access to resources Organizational level: location/accessibility and competing demands or mandates		X	X		X		X	
Recruitment logistics	- Focus on health improvement in recruitment instead of cardiovascular disease prevention - Using trusted individuals to recruit workers - Making eligibility criteria less restrictive for classes or exercise programming	Tailoring/tailoring the recruitment tools or plans to improve acceptability among workers	Increase reach or engagement Recipient level: motivation and readiness	X		X				X	
Retention plans	- Use of appropriate channels to communicate with participants - Provide short-form make-up classes - Be flexible with make-up classes - Provide additional incentives	Tailoring, adding elements, and shortening/improve and support participant engagement	Increase retention Recipient level: motivation and readiness and access to resources Organizational level: competing demands or mandates and available resources		X			X	X	X	X

Headers correspond to the following FRAME domains: ^a^What is the nature of the adaptation and why was it done?; ^b^What is the goal of and reason for the adaptation?; ^c^When did the adaptations occur?; ^d^Where modifications planned?; ^e^Who participated in the decision to make the adaptation? Here, reported as specific players; in FRAME, these groups are included in the following categories: INDIA-WORKS staff includes treatment/intervention team, program developers and purveyors, and individual practitioners/facilitators; peer educators are individual practitioners/facilitators; and worksite managers are administration

**Table 3:** Organization-level adaptations to the INDIA-WORKS program

**Description**	**Examples**	**Nature**^**a**^**/additional detail**	**Goal** **Reason**^**b**^	**Timing**^**c**^		**Planned**^**d**^			**Players**^**e**^		
				**Pre-implementation**	**Implementation**	**Planned/proactive**	**Planned/reactive**	**Unplanned/reactive**	**INDIA-WORKS staff**	**Worksite managers**	**Peer educators**
Worksite exercise facilities	- Allowing use of worksite floor/grounds for walking - Creation of new walking trails or exercise spaces	Adding elements	Improve effectiveness/outcomes Organization: available resources and mission	X	X	X		X		X	
Worksite canteen	- Offer more healthy choices at the canteen - Update recipes to be healthier - Create and post posters with information on healthy eating behaviors	Adding elements: offering and promoting healthier food options to the workforce	Improve effectiveness/outcomes Organization: available resources and mission	X		X				X	
Health programming at the worksite	- Invite INDIA-WORKS team members to do lectures on diabetes/ diabetes prevention - Use regular worksite health screenings to identify individuals for the intervention - Promote participant successes at team meetings	Adding elements: leveraging worksite events and team meetings to promote the program, provide opportunities for users, and improve participant success	Improve effectiveness/outcomes Organization: available resources and mission		X	X	X			X	

Headers correspond to the following FRAME domains: ^a^What is the nature of the adaptation and why was it done?; ^b^What is the goal of and reason for the adaptation?; ^c^When did the adaptations occur?; ^d^Where modifications planned?; ^e^Who participated in the decision to make the adaptation? Here, reported as specific players; in FRAME, these groups are included in the following categories: INDIA-WORKS staff includes treatment/intervention team, program developers and purveyors, and individual practitioners/facilitators; peer educators are individual practitioners/facilitators; and worksite managers are administration

### Adaptations to the individual-level intervention

Content adaptations affected the individual-level intervention and included changes to the program curriculum as well as tailoring program and testing logistics to make the program more understandable and approachable for program participants (Table 2).

#### Adaptations to the INDIA-WORKS curriculum

The most discussed adaptations were to the curriculum. Managers, peer educators, and INDIA-WORKS staff all discussed the importance of presenting the curriculum in a way that is understandable and relatable to the worksite employees. Adaptations included planned changes pre-intervention (e.g., translating to the local language) and reactive adaptations implemented during the program to improve program acceptability and participant engagement (e.g., reducing technical language). Peer educators and INDIA-WORKS staff tailored examples and dietary advice to local diets, focusing on eating smaller servings of foods that were familiar to participants to improve participant acceptability. A few peer educators also felt that local diets were the best suited for disease prevention and health. As one Peer Educator shared: “suppose I was born and brought up in a Chattisgarhi family and since my birth, whole time my three meals are only rice and rice and rice. This is never going to harm me … your body is adjusted to that type of diet”. A couple of peer educators made additional adaptations in the lifestyle classes they led based on personal views; for example, adding breathing exercises to reduce stress or moving the order of lessons to focus first on physical activity then on diet. This last example was not consistent with the class curriculum, reflecting a lack of fidelity to the program.

Several sites expanded the exercise program during implementation to add more advanced options like more intensive aerobics, Zumba, or yoga classes. Zumba classes were particularly well received by INDIA-WORKS participants (“Everyone likes to dance, but no one wants to do exercise. But when it is merged into one, everyone is interested”. Peer Educator). Some worksites refined the physical activity curriculum to provide additional training on skills they felt participants were lacking. This included helping participants to dress in proper exercise clothing and shoes for comfort and safety, focusing on proper breathing techniques when exercising, proper stretching to decrease pain and soreness, and being more active in their daily lives.

#### Adaptations to program and testing logistics

To increase overall program acceptability, sites adapted the program to improve testing uptake, recruitment, and retention. INDIA-WORKS staff members reported applying lessons learned at initial screening and testing visits to testing at subsequent worksites (unplanned, reactive adaptation); for example, collecting blood samples and follow-up data at a single study visit instead of multiple visits improved data completion. One worksite expanded study testing to better monitor program success by funding HbA1c and exercise testing for all participants.

Worksites sought to improve enrollment and retention by making the classes appealing to employees. Classes were held at times convenient for participants and permissible to supervisors. Classes at some sites were held during work hours, but most worksites (“90 percent” INDIA-WORKS staff) held classes after work hours. When this was challenging for participants, some worksites agreed to allow participants 30 min at the end of the workday for classes. At sites with shift workers, class times changed as participants’ shifts changed. INDIA-WORKS staff worked with management to select the most convenient venue for classes to improve participation and noted that different locations yielded better, or worse, class attendance. Although class scheduling was a planned adaptation, worksites had to continue making reactive/unplanned changed to class times and locations to find the ideal class time for different workers at the site.

Other sites sought to increase enrollment proactively by making the program more appealing to participants or widening eligibility criteria for some or all intervention components. One manager described how the program was presented as a way to reduce risk factors instead of labeling participants: “When you tell the participants…‘you are a pre-diabetic, you are a hypertensive, you are a diabetic’, they feel offended. They feel bad. They feel depressed. …we say, “You are a high-risk individual. There is goal for improvement”. (Worksite Manager). Another worksite hired retired local doctors to promote the program. Other sites allowed non-eligible workers to participate in some program components (e.g., lifestyle or exercise classes) but not study testing.

During program implementation, worksites made reactive adaptations to improve participant retention. Across sites, peer educators and INDIA-WORKS staff, and at some worksites managers, reported calling or messaging (via text or WhatsApp) participants to remind them of class, schedule sessions, or reschedule makeup sessions when conflicts arose. Makeup sessions, either newly scheduled sessions, one-on-one classes, or allowing participants to join another group for that week, were commonly employed to ensure that participants covered the course material even when their work schedules changed or required them to miss a class. For workers facing the most barriers (see Challenges to Adaptation), lessons were delivered individually at participants’ desks.

### Adaptations to the environmental-level intervention

Organization-level adaptations are shown in Table 3. While changes to the worksite canteens were all planned/proactive and pre-implementation, worksite-level changes to physical activity resources and health programming were both planned and unplanned and occurred both pre- and during implementation. Managers reported that making environmental changes at the worksite required them to consider both health recommendations and worker preferences to increase acceptability and avoid push-back. For example, one manager described how canteen changes needed a balanced approach instead of drastic changes: “Simply only giving diet biscuit… people will become mad” and less healthy foods (e.g., fried foods) need to be offered “once in a while”. A few sites created new print materials (e.g., posters) to reinforce the messages taught in the INDIA-WORKS classes (reading nutrition labels, proper portion sizes) and posted them in common areas. Another site provided nutrition information for canteen foods.

Many sites leveraged existing health promotion programming to provide INDIA-WORKS participants and other workers guidance on healthy lifestyles, disease prevention, and disease management. Several sites regularly invited experts (including INDIA-WORKS investigators) to present on health-related topics. Other sites discussed health issues or reviewed worker health statistics at team meetings. One worksite has an extensive health library including digital educational tools and a health educator available to all workers as well as a health statistician, psychologist, and other health providers on staff. One worksite, an occupational health industry, prioritized health programming (twice monthly screening camps, regular health talks, quarterly diabetes camps); INDIA-WORKS was easy to integrate and peer educators felt their roles in the INDIA-WORKS program were “part of job. That’s all. See we are here to prevent”. (Peer Educator).

### Training program-level adaptations

Most worksites delivered the INDIA-WORKS training program for peer educators without adaptations. Only one worksite hosted additional trainings sessions for peer educators to help them master the material and improve comfort with teaching. At all sites, INDIA-WORKS staff providing on-going support and training. One INDIA-WORKS staff shared: “This is a completely new forte for them. I won’t be able to do their job if I am asked to. I might do it if you explain to me well enough... Some [peer educators] would even come to our rooms and ask us how to present a certain thing etc. We gave individual attention to everyone”.

### Challenges to program adaptation

Scheduling (and rescheduling) classes around participant schedules, travel time to and around the worksite, production deadlines, and shift work was a “Herculean task” (Worksite Manager) and was discussed more frequently than any other adaptation barrier and across interview participant type. Similarly, sites with multiple worksites or a diversity of employee roles/positions found it challenging to find convenient schedules for classes and noted that program champions and staff had to gain support from management at each site or unit.

Interviewees also noted that the teaching ability, confidence to lead, and dedication of the peer educators varied greatly across sites. For example, at one site, peer educators often did not turn up for classes they were assigned to lead, leaving INDIA-WORKS staff to coach participants, while at another worksite, peer educators engaged in teaching classes and leveraged the INDIA-WORKS staff to provide assistance with more complex lessons and tailor class scripts to the health literacy and education level of workers in their classes.

Concurrent with INDIA-WORKS (but starting before COVID-19-related disruptions), several worksites were affected by major organization-level challenges such as privatization and a company shut down, which made it more difficult to adapt and deliver the INDIA-WORKS program. These challenges resulted in a lack of funds for implementation of environmental-level interventions like canteen changes and difficulty prioritizing the individual-level intervention for managers and participants. In addition, workers’ reactions to these challenges affected how peer educators, INDIA-WORKS staff, and managers were able to adapt the program at their worksites. For example, at one worksite where plans to privatize the worksite were being implemented, some employees at the site blamed the walking patterns of the INDIA-WORKS participants:

*[INDIA-WORKS participants] were regularly doing the exercises by walking around the building. Once the privatization issue came up, people started blaming the lack of orders on this walking routine around the building. So the person in charge told me to avoid walking around the building and to walk on one side instead. This was based on the superstition that if one walks around a building, it causes a negative effect. So that caused people to lose their heart a little.* INDIA-WORKS staff

### Suggestions for future program adaptations

Interview participants suggested additional content adaptations to consider in the future to improve sustainability and scalability of the lifestyle change program. Participants felt continued adaptations would be beneficial including timing and location of classes, variety of exercise types, and widening inclusion criteria. Interviewees suggested expanding the curriculum content by including additional diet and activity advice, information on worksite specific hazards (e.g., noise pollution, dust), and more audio-visual materials. Peer educators, staff, and managers suggested that the class scripts will need periodic updates to make them fresh and appealing to workers: “things keep on changing. So, you should also keep on changing”. (Worksite Manager).

Participants were conflicted about optimal timing and length for the program. Several peer educators and INDIA-WORKS completers suggested that longer or more frequent class periods were needed to cover all the material, provide individual feedback to participants, and do exercise classes, which were especially well received by people in their cohorts. Other interview participants suggested a shorter program (e.g., less or shorter sessions, half/full day classes taught over two days, monthly classes); these interviewees felt that shorter classes would improve participants’ attention and learning: “They come. They sleep. They fiddle the phone. You know attention span is maximum fifteen twenty minutes.… And in this small session, is only a small gathering. so, everybody is interactive”. (Worksite Manager).

Other suggestions mentioned by peer educators and INDIA-WORKS staff focused on creating an environment that enabled Peer Educator success. Suggestions included scheduling classes around peer educators’ time first and expanding the training program for peer educators to include more role playing, visual examples, and additional training on lecturing/teaching to help them feel better prepared to lead classes.

## Discussion

Integrated innovation theory posits that in order for an intervention to be successful in complex situations like community settings, it must integrate scientific/technological, social, and business innovations [39]. INDIA-WORKS fulfills this requirement: It delivers scientific innovations (proven lifestyle change education programs with text message supports during maintenance) with social innovations (trained peer health educators delivering a program to a large at risk population) and business innovation (worksite stakeholder commitment and partnering researchers to help deliver the program with fidelity, improve the workplace health environment, and evaluate the model). The packaging of individual lifestyle education with environmental changes at the worksite level, implemented through an academic-industry partnership, is rarely done, particularly in India, a population with acutely high risk for diabetes and diabetes-related complications. If successful, INDIA-WORKS could provide a model for innovative delivery of lifestyle education.

INDIA-WORKS program adaptations included changes to the curriculum, recruitment or retention plans, and creation or use of health programming and resources at the worksites. The majority of adaptations were done during implementation to respond to the needs of the participants and worksite, although adaptations like language translation and changes to worksite exercise and food environments where adapted pre-implementation. Most adaptations were planned, reflecting the large number adaptable components in INDIA-WORKS. Unplanned adaptations occurred in reaction to unanticipated challenges with study recruitment, retention, and classroom logistics due to worksite-specific barriers or challenges. Other unplanned adaptations, such as adding additional cultural adaptation to diet lessons or new types of physical activity, where made in reaction to requests and interests of program participants. Participants’ requesting additional physical activity options might reflect improvements in fitness, as increased fitness can lead individuals to engage in a wider variety of exercise types [40].

Except in one case (where a peer educator changed the order of lessons), adaptations were fidelity-consistent. This may have occurred because the program was carefully designed and presented to study partners with clearly defined core components, those necessary to program fidelity and aligned with proven behavioral health theories or strongly associated with program success in prior studies, and adaptable components which could be changed to be responsive and appropriate to the needs of the individual worksites [41]. This is vital to program success because a program that has been changed in a way that affects components associated with efficacy is doomed to fail even if delivered at the highest quality and consistency [42].

All key players in overseeing program implementation were empowered to adapt program components. Adaptations by peer educators involved the program curriculum or participant retention, items which interfaced directly with participants, while INDIA-WORKS staff focused on adaptations necessary to the conduct of the study at each setting including tailoring of curriculum and recruitment methods to best suit the worksite environment and workers. Manager-led adaptations were often in areas requiring higher-level approvals and coordination (e.g., changes in the canteen, deciding when and where lifestyle classes could be held). In Weiner, Lewis, and Linnan’s theory of the organizational determinants of effective program implementation at worksites, the authors argue that the complexity of implementing projects at worksites require active participation by both management and employees [42]. Other studies support this by showing that buy-in from management and employees was key to worksite program success or lack thereof [43–45].

Although, delivering a diabetes prevention program at worksites in India at no monetary cost to the employee can overcome many of the barriers to lifestyle change (e.g., cost of classes, lack of time, inability to locate acceptable resources for weight loss), interview participants still described barriers to adapting the program to the worksites. These included unanticipated worksite changes and disruptions, the ongoing challenge of scheduling and rescheduling classes, and diversity of employees, which have been reported as barriers to worksite health programming in other studies [44, 46, 47]. Suggestions for future program adaptations sought to overcome these challenges and often expanded on the themes identified and adapted during the INDIA-WORKS study. Interview participants shared the need to continue adapting and expanding the program to add interest and variety, better respond to worksite specific challenges, and enable more workers to participate in the program.

This study adds to the limited literature using the FRAME to describe program adaptations and modifications in the Indian context. Although the FRAME was able to categorize and describe most modifications with sufficient detail and clarity, there were some challenges to using the framework. Although various players were involved in suggesting program adaptations, implementation of adaptations always involved the worksite managers, with curriculum changes being the exception (those leading classes or trainings, peer educators and INDIA-WORKS staff, were able to make these adjustments independently). India remains strongly hierarchal, and worksites reflect this top-down model [48]. Managers expect to make the final decisions and workers defer to supervisors. Because of this, the FRAME domain “who made the decision to modify” may not accurately collect information on those involved in the decision to make modifications, since the final choice was often made at the management level. Furthermore, because of this hierarchy, it is possible that some participants were reluctant to report adaptations that were made but not approved by management, meaning that some modifications might not have been captured.

There was also additional challenges in categorizing some of the modifications because of lack of clarity with definitions in the FRAME documentation. For example, we categorized adaptations to program recruiting and retention activities and scheduling of classes as content modifications because they related to the way the treatment (e.g., intervention activities including screening and testing) was delivered. Furthermore, these adaptations were not related to any of the categories of contextual modifications described in the FRAME (format, setting, personnel, or population). In practice, use of the FRAME to document and monitor program adaptations by worksites would be even more challenging, given lack of training and experience in implementation sciences.

Finally, it was both time and resource intensive to document and categorize adaptations in detail and over time. To ensure we understood the full extent of adaptations, we conducted interviews with an array of key players involved in program implementation, and we continued interviews until data saturation was met. Each interview was then transcribed and coded, and thick descriptions were developed before categorizing the data using FRAME. This work was feasible given that this was a large, National Institutes of Health-funded study; however, less resourced study teams or community-based evaluators may not be able to commit to work of this scope to properly document program modifications and adaptations, leading to additional gaps in the literature on real-world implementation of health programs. Additional methodological development is needed to understand how to efficiently document program adaptations on an ongoing basis and how to best use this information to continuously improve interventions during implementation.

This manuscript describing adaptations to the INDIA-WORKS intervention program has several notable strengths. Although there is tremendous growth in the number of community-based interventions, reporting of program adaptations is still limited and often done non-systematically [49, 50]. Adaptations at INDIA-WORKS worksites were driven by individuals working at the site or doing in-field implementation work, instead of researchers, allowing a real-world assessment of adaptations at the study sites. In addition, although India has the second-largest workforce in the world [51], there is limited literature on worksite-based wellness programs in this setting, which is particularly important given the size and diversity of the workforce [52] as well as the large and growing number of individuals at working ages who are affected by cardiometabolic diseases in India [53–55]. The application of the FRAME allows for systematic reporting of the adaptations as well as enabling comparison with other studies of intervention adaptations.

However, the FRAME did not guide data collection. Instead, the framework was applied during data analysis to capture information on modifications emerging in the data. This could result in missing data including not all modifications being identified or fully characterized. Similarly, using IDIs only to track adaptations might lead to underreporting of program changes. Future studies of this type should consider using the FRAME and mixed methods data collection systematically to describe and track adaptations in real time. Similarly, although we interviewed a variety of stakeholders involved in the program implementation, including program delivery personnel, managers, and participants, the qualitative data collection was not focused primarily on understanding adaptations, and all worksite-specific changes might not have been captured.

Lastly, we did not explore how specific modifications and adaptations influenced implementation success and intervention effectiveness. The COVID-19 pandemic precluded further qualitative data collection at sites and made collection of additional information from the already-overburdened worksites practically impossible (e.g., adding new worksheets for managers to document additional changes was determined to be too challenging given that managers were already dealing with adapting the worksites to protect workers from COVID-19 transmission). Future studies should evaluate various adaptations for improving participation, retention, program delivery, and indicators of program effectiveness like weight loss. However, there is still great value in reporting program modifications. Very little is known about how programs are adapted in general and in low -and middle-income countries and worksites, specifically. This study goes beyond simply describing planned program methods to provide important guidance on how the program was delivered at individual worksites. This information can be used to help with program scaling and guide other researchers and program planners designing similar programs. Documenting unplanned modifications is particularly important as they can be proactively considered in the future to ensure all adaptation are fidelity consistent. Similarly, this work can be used to help with the design and planning of worksheets and other documentation for recording adaptations and provides documentation to assist in manualizing the intervention for broader dissemination (e.g., examples of adaptable elements, forms for documenting program fidelity).

## Conclusion

In conclusion, this manuscript describes and characterizes, using the FRAME classification system, worksite specific adaptations to the INDIA-WORKS intervention. Sites reported adaptations to the curriculum, participant recruitment and retention, and worksite health resources. This study adds to the growing literature reporting on program adaptations in implementation research in general and is one of the first to specifically describe implementation of lifestyle programs for early identification of non-communicable disease and prevention of cardiometabolic disorders at worksites in India.

## Data Availability

The dataset (de-identified transcripts of the IDIs and FGDs) analyzed during the current study are available from the corresponding author on reasonable request.

## Abbreviations

BMI: Body mass index
D-CLIP: Diabetes Community Lifestyle Improvement Program
FGD: Focus group discussion
FRAME: Framework for Reporting Adaptations and Modifications-Enhanced
HbA1c: Glycated hemoglobin
IDI: In-depth Interview
INDIA-WORKS: Integrating Diabetes Prevention in Workplaces
